# Effects of Coronavirus (COVID-19) Fear on Oral Health Status

**DOI:** 10.3290/j.ohpd.b1870377

**Published:** 2021-09-11

**Authors:** Aysegul Sari, Zuhal Yildirim Bilmez

**Affiliations:** a Assistant Professor, Faculty of Dentistry, Department of Periodontology, Hatay Mustafa Kemal University, Hatay, Turkey. Study concept and design, designed questionnaire, collected data, wrote, edited, and approved the final manuscript.; b Assistant Professor, Department of Restorative Dentistry, Faculty of Dentistry, Hatay Mustafa Kemal University, Hatay, Turkey. Study concept and design, designed questionnaire, collected data, wrote, edited, and approved the final manuscript.

**Keywords:** Coronavirus, dental diseases, fear, habits, oral health

## Abstract

**Purpose:**

To investigate the effects of COVID-19 fear on oral health status.

**Materials and Methods:**

A total of 1227 participants were enrolled in the study. The online survey link was circulated and responses were received. The questionnaire comprised a total of 24 closed-ended questions, which were divided into three sections. The first section focused on demographic information, the second section on the fear of COVID-19, and the third section focused on oral hygiene habits, dental complaints, and tendency to go to the dentist during the COVID-19 pandemic.

**Results:**

Participants who had a higher fear score compared to other respective populations during the corona virus pandemic started brushing more regularly, started to use oral care products more regularly (p = 0.001), and reported increases in: sugary food consumption (p = 0.001), meat consumption (p = 0.017), vegetable consumption (p = 0.019), tooth hypersensitivity, toothache, swelling/abscess on face due to tooth decay (p = 0.001), restoration failures, problems with prostheses (p = 0.007), bleeding and pain in the periodontal tissue, oral malodor, and bruxism (p = 0.001). They also had dental problems but hesitated to visit a dentist, and thought that dental clinics are at risk of COVID-19 contamination (p = 0.001).

**Conclusion:**

Fear of COVID-19 was higher in participants who started to pay more attention to their oral hygiene habits, had a change in food consumption frequency and rate, had an increase in oral and dental health complaints, and had dental problems but hesitated to visit a dentist.

A new coronavirus causing pneumonia in humans was identified in Wuhan, China in 2019 and named SARS CoV-2 due to its taxonomic similarity with severe acute respiratory syndrome (SARS).^51^ The number of people infected with this novel coronavirus increased rapidly and spread across continents. As a result, coronavirus disease 2019 (COVID-19) was declared a pandemic by the World Health Organization (WHO) on March 11, 2020.^54^

The transmission route of the virus is via aerosols and contact. It is rapidly spread and highly contagiousness, especially through small aerosols from the nose and mouth that are expelled when an infected person coughs, sneezes, or speaks.^26^ Approximately 80% of infected people are asymptomatic, 5% are critical infections that need intensive care, and asymptomatic people can likewise infect others.^42^ For this reason, various measures, such as restriction of social life and quarantine, have been taken in many countries to prevent the spread of the virus during the COVID-19 pandemic. Measures that restrict the occupational and educational lives of individuals and limit their social contact with each other have led to a decrease in the quality of life and increased important problems such as fear, anxiety, and anxiety disorders.^34^ There are studies reporting an increase in anxiety in the population due to the increasing number of infected people and relatively high mortality. In particular, there is a fear of contacting people possibly infected with COVID-19.^19,27^ It is known that fear is associated with disease transmission, morbidity, and mortality rates.^37^ As of December 29, 2020, the WHO reported that the total number of deaths due to COVID-19 was 1.7 million and 79 million confirmed cases of COVID-19 in 2020 globally.^53^ People who changed their daily routines and activities in addition to fear and anxiety due to the COVID-19 pandemic try to cope with the effects of the process by exhibiting different behavioural reactions, such as taking their oral care habits more seriously or by becoming negligent.

The changes in individuals’ lives affected their habits and daily routines. Changes in sleeping patterns and nutritional content have occurred. This situation can lead to changes in an individual’s general health as well as oral and dental health. It can also cause an increase in food intake, including overprocessed and high-calorie foods.^12-32^ High-carbohydrate diets can lead to various health problems, such as diabetes, obesity, and poor oral health.^11-33^ A study reported that the intake of red meat, sugary drinks and potato chips increased during quarantine.^39^ Frequent consumption of sugar can increase biofilm accumulation on the tooth surface and cause the formation of carious lesions and periodontal disease.^11^ In addition, routine changes may cause delays in daily oral and dental care.^17^ A study on children reported that the psychological effects of the pandemic are associated with changes in oral hygiene routines and eating patterns.^10^

Fear of COVID-19 can increase stress, instability, and economic anxiety, leading to a decrease in overall well-being during the pandemic period. It has been reported that high levels of fear can produce negative health consequences and cause physical and emotional damage.^8^ A study^38^ has suggested that delaying dental treatments due to fear of COVID-19 could increase the risk of serious health problems in the future. Those authors showed that the quarantine recommended due to the COVID-19 pandemic had an effect on dental appointments and patients’ anxiety, as there is a statistically significant relationship between patients’ emotions and their willingness to attend a dental appointment. In particular, patients who are anxious and afraid tend to visit dental clinics only for emergency dental treatments.^38^ In another study,^10^ individuals in regions where infection is common reduced their numbers of dental appointments because they thought it was unsafe. The level of fear in individuals in need of dental treatment was also associated with the number of individuals contaminated with COVID-19 in their local area.^10^ The negative effects of the Covid-19 fear on preventive and curative oral care services may expose inadequacies in the oral and dental health of society in the long term. For this reason, it can help formulate targeted intervention strategies to identify the groups most at risk in terms of COVID-19 fear and oral health and to ensure their optimum health.^10^

Most studies have focused on the clinical features and treatment of COVID-19 or the precautions necessary to prevent it. However, no study has evaluated the psychological changes caused by people’s fear of COVID-19 and the effects of fear on health behaviours. COVID-19 fear may affect oral health status by changing routine diet and oral care habits. At the same time, it may affect dental appointments. Considering all this, the outcomes of Covid-19 fear can negatively affect oral health status. Hence, the aim of the present study was to investigate the effects of COVID-19 fear on oral health status.

## Materials and Methods

### Study Population

The study was conducted in full accordance with ethical principles, including the World Medical Association’s Declaration of Helsinki as revised in 2008, and was approved by the Ethics Committee (protocol number 2020/16), Hatay Mustafa Kemal University, Hatay, Turkey.

The present cross-sectional study conformed to the Strengthening the Reporting of Observational Studies in Epidemiology (STROBE) guidelines for observational studies.^50^ The study was conducted using an online survey questionnaire, which was designed by www.googleforms.com, from August 1 to October 1, 2020. The online survey link was sent via e-mail and WhatsApp to participants and received responses through an online survey submission platform. The survey was conducted in Turkish and in Turkey. The questionnaire had a total of 24 mandatory closed-ended questions, which were divided into three sections. The first section focused on demographic information (8 questions), the second section focused on the fear of COVID-19 (7 questions), and the third section focused on oral hygiene habits, changing nutrition, dental complaints, and tendency to visit the dentist during the COVID-19 pandemic (9 questions: 23 items). This set of questions, except the COVID-19 scale, was designed by the authors, and examined by a periodontologist, an oral radiologist, a restorative dentist, and a biostatistician. Participation was voluntary, and the return of completed forms signified informed consent. All the questions had to be answered for the survey to reach the researchers.

The seven-item Fear of COVID-19 Scale was developed by Ahorsu et al in 2020.^2^ It consists of seven items and is used to assess fear regarding COVID-19. The participants indicated their level of agreement with the statements using a five-item Likert-type scale. The minimum score possible for each question is 1, and the maximum is 5. Answers included 1: strongly disagree; 2: disagree; 3. neither agree nor disagree; 4: agree; and 5: strongly agree. The total score was calculated by adding each item score (ranging from 7 to 35). The higher the score, the greater the fear of COVID-19.^2^ The Turkish version of the Fear of COVID-19 Scale was used in the study. Turkish validity and reliability of the scale was performed by Haktanir et al.^21^

A statistical power calculation was conducted for this study. The necessary sample size was 1.138 according to a standard deviation of 5.68 and a standard error of 0.33,^14^ taking into account that the population size was unknown.

Sample size calculation was performed using the following formula:^46^


$$n={\left(\frac{z\sigma }{E}\right)}^{2}$$

A total of 1227 individuals who answered the web-based questionnaire during the survey performance period were included in this study. The inclusion criterion of the questionnaire was age > 18 years and < 65 years.

### Statistical Analysis

Data distribution normality was tested using the Shapiro-Wilk test. Student’s t-test was used to compare normally distributed variables in two independent groups, and the Mann-Whitney U-test was used to compare non-normally distributed features in two independent groups. In addition, for the comparison of numerical data in more than two independent groups, one-way ANOVA and LSD multiple-comparisons tests were used for variables with normal distribution. The Kruskal-Wallis test and all pairwise multiple-comparisons tests were used for non-normally distributed variables. Relationships between numerical variables were tested using the Spearman correlation coefficient. Cronbach alpha coefficients were calculated to test the validity and reliability. As descriptive statistics, the mean ± standard deviation for numerical variables, and number and % values for categorical variables, are reported. Scores were categorised as low and high levels of fear based on the mean. The mean value was taken as a cutoff. Scores less than or equal to the mean were considered low fear, and scores above the mean were considered high fear.^14^ A comparison of low and high levels of fear and conditional stepwise logistic regression analysis of fear levels with all variables were conducted. Variables were selected using a modified backward stepwise process.

The statistical analyses were conducted using commercially available statistical software (SPSS v 24.0; IBM; Armonk, NY, USA), with statistical significance set at p<0.05.

## Results

### Demographic Findings

Demographic variables of the study participants, changing oral hygiene habits and nutrition, dental complaints, and tendency to go to the dentist are reported in Table 1. The study population comprised 695 (56.6%) females; the age range of 340 participants (27.7%) varied between 18 and 25.

**Table 1:** Characteristics of the study population, changing oral hygiene habits, nutrition, dental complaints, and tendency to go to the dentist

Variable			N (%)
Gender		Male Female	532 (43.4) 695 (56.6)
Age groups (years)		18-25 26-35 36-45 46-55 56-65	340 (27.7) 250 (20.4) 267 (21.8) 232 (18.9) 138 (11.2)
Marital status		Unmarried/single Married	490 (39.9) 737 (60.1)
Educational status		Elementary school High school University Master/PhD	79 (6.4) 214 (17.4) 771 (62.4) 163 (13.3)
Residence		City center District Village	732 (59.7) 421 (34.3) 74 (6)
Monthly income		2500 ₺ (Low income) 2500-5000 ₺ (average income) 5000 ₺ (high income)	182 (14,8) 430 (35) 615 (50.2)
How has your smoking habit changed during the pandemic?		I was smoking, but quit due to COVID-19 I was smoking, but reduced it due to COVID-19 Unchanged I was smoking, and increased it during the pandemic process Non-smoker	26 (2.1) 96 (7.8) 167 (13.6) 57 (4.6) 881 (71.8)
Do you have any chronic diseases?		No Yes	915 (74.6) 312 (25.4)
How has your brushing habit changed during the pandemic?		I have started brushing more regularly My brushing routine has been disrupted There has been no change in my brushing pattern	505 (41.1) 398 (32.49) 324 (26.41)
If there has been a change in your brushing habit, what is the reason?		I paid more attention to my oral hygiene to reduce the risk of virus contamination I’m very stressed because my daily routine has changed My sleep pattern is disrupted	531 (58.8) 218 (24.1) 154 (17.1)
Has there been a change in your frequency of using oral care products, such as dental floss, interdental brush, and mouthwash?		I started using it more regularly I started using it more irregularly Unchanged	121 (9.9) 89 (7.3) 1017 (82.9)
Has your eating frequency changed during the pandemic period?		I eat less often I eat more often Not changed	129 (10.5) 359 (29.3) 739 (60.2)
How has the frequency of your consumption of the following foods changed during the pandemic?	Sugary foods such as cakes and cookies Bakery foods such as bread and pastry Acidic drinks Meat and dairy products Vegetables Fruits	Has increased Has decreased Unchanged Has increased Has decreased Unchanged Has increased Has decreased Unchanged Has increased Has decreased Unchanged Has increased Has decreased Unchanged Has increased Has decreased Unchanged	408 (33.3) 180 (14.7) 639 (52.1) 430(35) 202 (16.5) 595 (48.5) 222 (18.1) 278 (22.7) 727 (59.3) 426 (34.7) 73 (5.9) 728 (59.3) 426 (34.9) 111 (9) 688 (56.1) 481 (39.2) 92 (7.5) 654 (53.3)
How have your oral and dental health complaints changed during the pandemic?	Sensitivity due to cold and sugary foods Toothache due to hot foods Swelling /abscess on my face due to tooth decay Failure of restorations Problems with prostheses Injuries or fractures in the tooth, jaw, or face area Bleeding in periodontal tissue Pain in the periodontal tissue Oral malodor Bruxism	Has increased Has decreased Unchanged Has increased Has decreased Unchanged Has increased Has decreased Unchanged Has increased Has decreased Unchanged Has increased Has decreased Unchanged Has increased Has decreased Unchanged Has increased Has decreased Unchanged Has increased Has decreased Unchanged Has increased Has decreased Unchanged Has increased Has decreased Unchanged	145 (11.8) 78 (6.4) 1004 (81.8) 82 (6.7) 69 (5.6) 1076 (87.7) 38 (3.1) 73 (5.9) 1116 (91) 76 (6.2) 70 (5.7) 1081 (88.1) 68 (5.59) 64 (5.2) 1095 (89.2) 33 (2.7) 67 (5.5) 1127 (91.9) 127 (10.4) 92 (7.5) 1008 (82.2) 124 (10.1) 86 (7) 1017 (82.9) 108 (8.8) 117 (9.5) 1002 (81.7) 163 (13.3) 132 (10.8) 932 (76)
Have you consulted a dentist during the pandemic process due to your oral and dental problems?	I have a problem but I did not go to the dentist because I was afraid of disease I have a problem and went to the dentist for treatment I have no discomfort		328 (26.7) 146 (11.9) 753 (61.4)
Compared to the pre-COVID-19 period, have your thoughts changed about going to the dentist during the pandemic period?	I do not hesitate to go to the dentist compared to before COVID-19 I hesitate to go to the dentist compared to before COVİD-19 There was no change		89 (7.3) 619 (50.4) 519 (42.3)
Do you think dental clinics pose a risk of COVID-19 contamination?	Yes No I am undecided		927 (75.6) 94 (7.7.) 206 (16.8)

In this study population, marital status “married” (60.1%), educational status “university” (62.4%), and residence “city center”were common (59.75%). The most common monthly income was 5000 ₺ (high income) (50.2%), the most common smoking status was nonsmoker (71.8%), and systemic health status was most commonly systemically healthy (74.6%).

A total of 41.1% of the participants stated that they started brushing their teeth more regularly, and 32.5% reported that their brushing routine was disrupted. A total of 58.8% of the participants changed their brushing habits because they paid more attention to oral hygiene to reduce the risk of virus contamination; 24.1% were stressed because daily routines had changed, and 17.1% had interrupted sleeping patterns. The percentage of participants who started using oral care products – e.g. dental floss, interdental brushes, and mouthwash – more regularly was 9.9%, while 7.3% started using the products more irregularly, and 82.9% reported no change.

Most of the participants stated that their eating patterns had not changed. A total of 29.3% of participants stated that they started eating more often. 33.3% of them stated that consumption of sugary foods such as cakes, cookies, and biscuits increased. 35% of them stated that consumption of bakery foods such as bread and pastry had increased. 34.7% of them stated that consumption of meat and dairy products had increased, 34.9% stated that consumption of vegetables had increased, 39.2% of them stated that consumption of fruits had increased.

Most of the participants stated that they had no change in oral and dental health complaints. A total of 11.8% stated that sensitivity to cold and sugary foods increased, 6.7% stated that toothache due to hot foods had increased, 6.2% stated that more restorations had failed. 10.4% of participants stated that bleeding in the periodontal tissue had increased, 10.1% reported that pain in the periodontal tissue had increased, 8.8% reported that oral malodor had increased, and 13.3% stated that bruxism had increased.

Regarding consultation with the dentist before and after COVID-19, 26.7% of the participants stated that they had a problem but did not go to the dentist due to fear of COVID-19, 11.9% had a problem and went to the dentist for treatment, and 61.4% had no discomfort.

50.4% of the participants stated that they hesitated to go to the dentist compared to before COVID-19. A total of 75.6% of them stated that they thought that dental clinics were at risk of COVID-19 contamination.

### Fear of COVID-19 Scale Findings

The item-wise responses of the Fear of COVID-19 Scale are shown in Table 2. The the item-wise means of responses were as follows: Most of the responses to items 1 and 2 were neither agree nor disagree nor agree (3.17 ± 1.30 and 3.29 ± 1.39, respectively); most of the responses to items 4 and 5 were neither agree nor disagree (2.56 ± 1.43 and 2.90 ± 1.34, respectively); most of the responses to items 3, 6, and 7 were disagree (1.68 ± 1.11, 1.54 ± 1, and 1.84 ± 1.21, respectively).

**Table 2:** Item-wise responses on the Fear of COVID-19 scale

Items	Median	Mean±SD
1. I am most afraid of COVID-19.	3	3.17 ± 1.30
2. It makes me uncomfortable to think about COVID-19.	3	3.29 ± 1.39
3. My hands become clammy when I think about COVID-19.	1	1.68 ± 1.11
4. I am afraid of losing my life because of COVID-19.	2	2.56 ± 1.43
5. When watching news and stories about COVID-19 on social media, I become nervous or anxious.	3	2.90 ± 1.34
6. I cannot sleep because I’m worried about getting COVID-19.	1	1.54 ± 1
7. My heart races or palpitates when I think about getting COVID-19.	1	1.84 ± 1.21
Total score	16	16.98 ± 6.59

Cronbach’s alpha for total scale scores = 0.868.

### The Fear of COVID-19 Scale Low and High Fear Score Comparison

On the Fear of COVID-19 scale, a comparison of low and high fear scores based on demographics, changing oral hygiene habits and nutrition, dental complaints, and tendency to visit the dentist are shown in Table 3. Participants who had a higher fear score compared to other respective populations during the coronavirus pandemic were: females (p = 0.001), married (p = 0.018), and a lower educational status (p = 0.033). Higher fear scores also existed among those who had chronic disease (p = 0.001), started brushing more regularly (p = 0.001), started to more regularly use oral care products such as dental floss, interdental brush, and mouthwash (p = 0.001). These participants had also increased their: eating frequency (p = 0.001), consumption of sugary foods (p = 0.001), bakery foods (p = 0.001), meat and dairy products (p = 0.017), and vegetables (p = 0.019). Participants with higher fear scores also had increases in: tooth hypersensitivity (p = 0.001), toothache (p = 0.001), swelling/abscess on the face due to tooth decay (p = 0.001), restoration failures (p = 0.001), problems with prostheses (p = 0.007), bleeding in the periodontal tissue (p = 0.001), pain in the periodontal tissue (p = 0.001), oral malodor (p = 0.001), and bruxism (p = 0.001). These participants also had dental problems but hesitated to visit a dentist (p = 0.001). They reported that they were more hesistant to visit the dentist compared to before the COVID-19 pandemic (p = 0.001), and thought that dental clinics were at risk of COVID-19 contamination (p = 0.001).

**Table 3:** The Fear of COVID-19 Scale, comparison of low and high fear scores based on demographics, changing oral hygiene habits and nutrition, dental complaints, and tendency to visit the dentist

Variable			Low N (%)	High N (%)	p
Gender		Male Female	335 (52.5) 303 (47.5)	197 (33.4) 392 (66.6)	0.001
Age groups (years)		18-25 26-35 36-45 46-55 56-65	190 (29.8) 131 (20.5) 134 (21) 120 (18.8) 63 (9.9)	150 (25.5) 119 (20.2) 133 (22.6) 112 (19) 75 (12.7)	0.324
Marital status		Unmarried/single Married	275 (43.1) 363 (56.9)	215(36.5) 373 (63.5)	0.018
Educational status		Elementary school High school University Master/PhD	32 (5) 101(15.8) 411 (64.4) 94 (14.7)	47 (8) 113 (19.2) 360 (61.1) 69 (11.7)	0.032
Location of residence		City center District Village	3858(60.3) 211(33.1) 42(6.6)	347 (58.9) 210 (35.7) 32(5.4)	0.504
Monthly income		2500 ₺ (low income) 2500-5000 ₺ (average income) 5000 ₺ (high income)	95 (14.9) 214 (33.5) 329 (51.6)	87 (14.8) 216 (36.7) 286 (48.6)	0.493
How has your smoking habit changed during the pandemic?		I was smoking, but quit due to COVID-19 I was smoking, but reduced it due to COVID-19 Unchanged I was smoking, and my use increased during the pandemic process Non-smoker	11 (1.7) 51 (8) 91 14.3) 29 (4.5) 456 (71.5)	15 (2.5) 45 (7.6) 76 (12.9) 28 (4.8) 425 (72.2)	0.828
Do you have any chronic diseases?		No Yes	504 (79) 134 (21)	411 (69.8) 178 (30.2)	0.001
How has your brushing habit changed during the pandemic?		I have started brushing more regularly My brushing routine has been disrupted There has been no change in my brushing pattern	68 (10.7) 66 (10.3) 504 (79)	108 (18.3) 72 (12.2) 409 (69.4)	0.001
If there has been a change in your brushing habit, what is the reason?		I paid more attention to my oral hygiene to reduce the risk of virus contamination I’m very stressed because my daily routine has changed My sleep pattern is disrupted	272 (62) 92 (21) 758 (17.1)	259 (55.8) 126 (27.2) 79 (17)	0.081
Has there been a change in the frequency of using oral care products such as dental floss, interdental brush and mouthwash?		I started using them more regularly I started using them more irregularly Unchanged	43 (6.7) 33 (5.2) 562 (88.1)	78 (13.2) 56 (9.5) 455 (77.2)	0.001
Has your eating frequency changed during this pandemic period?		I eat less often I eat more often Unchanged	60 (89.4) 155 (24.3) 423 (66.3)	69 (11.7) 204 (34.6) 316 (53.7)	0.001
How has the frequency of your consumption of the following foods changed during the pandemic?	Sugary foods such as cakes, cookies, biscuits Bakery foods such as bread and pastry Acidic drinks Meat and dairy products Vegetables Fruit	Has increased Has decreased Unchanged Has increased Has decreased Unchanged Has increased Has decreased Unchanged Has increased Has decreased Unchanged Has increased Has decreased Unchanged Has increased Has decreased Unchanged	185 (29) 91 (14.3) 362 (56.7) 188 (29.5) 109 (17.1) 341 (53.4) 107 (16.8) 144 (22.6) 387 (60.7) 200 (31.3) 35 (5.5) 403 (63.2) 200 (31.3) 57 (8.9) 381 (59.7) 233 (36.5) 46 (7.2) 359 56.3)	223 (37.9) 89 (15.1) 277 (47) 242 (41.1) 93 (15.8) 254 (43.1) 115 (19.5) 134 (22.8) 340 (57.7) 226 (38.4) 38 (6.5) 325 (55.2) 228 (38.7) 54 (9.2) 307 (52.1) 248 (42.1) 46 (7.8) 295 (50.1)	0.001 0.001 0.421 0.017 0.019 0.092
How have your oral and dental health complaints changed during the pandemic?	Sensitivity due to cold and sugar foods Toothache due to hot foods Swelling / abscess on my face due to tooth decay Failure of restorations Problems with prostheses Injuries or fractures in the tooth, jaw, or face area Bleeding in the periodontal tissue Pain in the periodontal tissue Oral malodor Bruxism	Has increased Has decreased Unchanged Has increased Has decreased Unchanged Has increased Has decreased Unchanged Has increased Has decreased Unchanged Have increased Have decreased Unchanged Have increased Have decreased Unchanged Has increased Has decreased Unchanged Has increased Has decreased Unchanged Has increased Has decreased Unchanged Has increased Has decreased Unchanged	48 (7.5) 33 (5.2) 557 (87.3) 26 (4.1) 26 (4.1) 586 (91.8) 14 (2.2) 25 (3.9) 599 (90.8) 29 (4.5) 30 (4.7) 579 (90.8) 25 (3.9) 27 (4.2) 586 (91.8) 16(2.5) 30 (4.7) 592(92.8) 49 (7.7) 37 (5.8) 552 (86.5) 44 (6.6) 37 (5.8) 557 (87.3) 42 (6.6) 47 (7.4) 549 (86.1) 53 (8.3) 57 (8.9) 528 (82.8)	97(16.5) 45 (7.6) 447 (75.9) 56 (9.59) 43 (7.3) 490 (83.2) 24 (4.1) 48 (8.1) 517 (87.8) 47 (8) 40 (6.8) 502 (85.2) 43 (7.3) 37 (6.3) 509 (86.4) 17 (2.9) 37 (6.3) 535 (90.8) 78 (13.2) 55 (9.3) 456 (77.4) 80 (13.6) 49 (8.3) 460 (78.1) 66 811.2) 70 (11.9) 453 (76.9) 110 (18.7) 75 (12.7) 404 (68.6)	0.001 0.001 0.001 0.010 0.007 0.429 0.001 0.001 0.001 0.001
Have you visited your dentist during the pandemic due to your oral and dental problems?	I have a problem but I did not go the dentist, due to fear of Covid-19 virus I have a problem and went to the dentist for treatment I have no discomfort		125 (19.7) 70 (11.1) 438 (69.2)	194 (33.2) 76 (13) 315 (53.8)	0.001
How have your thoughts changed about going to the dentist during pandemic period?	I do not hesitate to go to the dentist compared to before Covid-19 pandemic I hesitate to go to the dentist compared to before Covid-19 pandemic There was no change		48(7.5) 262 (41.1) 328 (51.4)	41 (7) 357 (60.6) 191 (322.4)	0.001
Do you think dental clinics are at risk for COVID-19 contamination?	Yes No I am indecisive		460 (72.1) 69 (10.8) 109 (17.1)	467 (79.3) 25 (4.2) 97 (16.5)	0.001

p-values were obtained from Exact and Pearson’s chi-squared tests.

The logistic regression model for dependent variables as well as the low and high Fear of COVID-19 scores (cutoff mean) are shown in Table 4.

**Table 4:** The logistic regression model for dependent variables of the Fear of COVID-19 scale, low and high scores (cut-off mean)

Variables		Beta	SE	p	OR	95% CI	
						Lower	Upper
Male					1		
Female		0.680	0.127	0.001	1.975	1.541	2.531
Unmarried/single					1		
Married		0.435	0.132	0.001	1.545	1.193	2.002
Master/PhD					1		
Elementary school		0.691	0.298	0.020	1.996	1.113	3.579
High school		0.550	0.226	0.015	1.733	1.113	2.697
University		0.212	0.188	0.259	1.237	0.855	1.788
Has there been a change in the frequency of using oral care products such as dental floss, interdental brush and mouthwash?	No change				1		
	More regularly	0.609	0.213	0.004	1.839	1.213	2.790
	Has been disrupted	0.460	0.254	0.070	1.585	0.963	2.607
How have your toothache complaints with hot foods changed during the pandemic?	No change				1		
	Has increased	0.511	0.268	0.045	1.668	1.001	2.820
	Has decreased	0.531	0.282	0.059	1.701	0.980	2.953
How have your bruxism complaints changed during the pandemic?	No change				1		
	Has increased	0.663	0.201	0.001	1.941	1.310	2.878
	Has decreased	0.222	0.211	0.292	1.249	0.826	1.888
Have you visited your dentist during the pandemic due to your oral and dental problems?	No change				1		
	Have not hesitated	0.158	0.247	0.521	1.172	0.722	1.900
	Have hesitated	0.666	0.132	0.001	1.946	1.502	2.522

SE: standard error. The beta-value was obtained from the LR model. The logistic regression model was built for the Fear of Coronavirus Scale. Model fit was tested using the goodness-of-fit chi-squared and the Hosmer-Lemeshow statistic. Variables were selected using a modified backward stepwise process.

Participants who displayed higher odds of having a high level of fear than their respective populations female (OR: 1.975; p = 0.01), married (OR: 1.545; p = 0.01), elementary and high school graduates (OR: 1.996 and 1.773, respectivley; p = 0.02 and 0.015, respectively), had started to brush their teeth more regularly and use oral care products, e.g. dental floss, interdental brushes, and mouthwash (OR: 1.839; p = 0.004). These participants also had: increased toothaches with hot food (OR: 1.668, p = 0.045), increased bruxism (OR: 1.941, p = 0.001), and hesitated to visit a dentist (OR: 1.946, p = 0.001).

## Discussion

The COVID-19 pandemic very seriously affects social life and, in every respect, may change the routine of our daily habits by its possible effects on our psychological health. To our knowledge, this study was among the first to investigate the effects of COVID-19 fear on oral health status by changing routine diets and oral care habits of people and preventing them from going to the dentist.

The findings of the present study showed that participants with higher fear scores started to pay more attention to their oral hygiene habits, changed the frequency of their food consumption, consumed some foods at changed rates, had increased oral and dental health complaints, had a dental problem but hesitated to visit a dentist, and thought that dental clinics had a risk of COVID-19 contamination.

The COVID-19 pandemic, by its nature, has increased fear worldwide.^12-27^ The emergence of mild anxiety is a natural response. It can encourage preventive and safeguarding behaviour.^18^ On the other hand, excessive anxiety can cause panic and lead to incorrect behaviour and decisions.^33^ Females, married individuals, those with lower educational status, and those with chronic diseases had higher fear scores than their respective populations during the coronavirus pandemic. Anxiety disorders were seen more frequently in women.^3^ Similar to previous studies, females had 1.975 times higher odds of fear of COVID-19 than males in our study (OR: 1.975; p = 0.01).^36,37^ Married participants had 1.545 times higher odds of fear scores than unmarried/single participants in our study (OR: 1.545; p = 0.01), consistent with the literature.^14^ This result may be caused by their concern about their family’s and children’s health. Doshi et al^14^ stated that in their study evaluating COVID-19 fear, a lower educational level would make it difficult for individuals to understand the process of the infection, and strictly stated hygiene rules would increase their fear of the pandemic.^14^ Participants who had less education showed higher fear scores in our study, in accordance with the literature. Compared to participants with a Master’s/PhD degree, participants whose highest educational level was elementary or high school had 1.996 times (OR: 1.996; p = 0.02) and 1.733 times (OR: 1.996; p = 0.02) higher odds of having a fear of COVID-19, respectively. To reduce the fear levels of this group, it may be important to provide information about the disease and precautions using more descriptive methods. Individuals with systemic diseases, such as cardiovascular diseases, severe obesity (BMI ≥ 40 kg/m^2^), and diabetes mellitus, are more likely to become infected. Their risk is also higher for complications and death from COVID-19. ^22,31,40^ Individuals with systemic diseases, who are more at risk due to COVID-19, can be expected to have higher rates of psychological problems, such as fear and anxiety. Similar to our study results, one study showed that people with diabetes had high levels of concern related to the COVID-19 pandemic.^23^

The oral cavity can serve as an important reservoir for pathogens in respiratory infections.^43^ Some studies have shown that the increased risk of complications and death in COVID-19 may be associated with oral biofilms and periodontal diseases, and that increasing oral hygiene can reduce the risk of complications.^15,41^ Furthermore, toothbrushing with toothpaste twice a day has been recommended for the elderly in nursing homes and for those at high risk of contracting COVID-19.^1^ Toothbrushing and gargling have also been recommended to physically remove accumulated viral nucleic acids.^52^ Our survey results showed that oral care habits were affected for different reasons in the pandemic process. Some individuals stated that they performed their oral care more regularly to reduce the risk of contamination, while others stated that their oral care habits changed because their daily routines changed. A total of 41.1% of the participants stated that brushing started more regularly, and 32.49% of brushing routines were disrupted. In our study, a total of 58.8% of the participants had changed their brushing habits because they paid more attention to oral hygiene to reduce the risk of virus contamination, 24.1% were stressed because their daily routines changed, and 17.1% had interrupted sleeping patterns. In our study, most of the participants stated that they had not changed their use of oral care products, but higher fear scores were seen in participants who started brushing more regularly and in those who started to use oral care products such as dental floss, interdental brushes, and mouthwash more regularly. In another study,^24^ it was reported that people increased the frequency of toothbrushing compared to before the pandemic; the reason stated was that participants thought there was a relationship between COVID-19 infection and oral hygiene, and that good oral hygiene would reduce the risk of infection. These results were consistent with our findings. On the other hand, 32.5% of our study participants stated that their brushing routine has been disrupted. The most important reason for reducing the frequency of toothbrushing could be social isolation and absence from the community.^24^

In our study, participants’ answers to the questions about their eating habits and complaints related to oral health during the pandemic process were mostly ‘no change’. The participants of the survey were mostly individuals with high economic and educational status. In addition, at the time of the survey on the web, the curve of the pandemic had flattened to a plateau, and the new normal order with social distance started to apply. This situation may have caused a difference in the situations of the participants between the pre-quarantine, quarantine, and post-quarantine periods. These factors may have caused the participants to give more ‘no change’ answers to the questions.

The quarantine period and COVID-19 outbreak caused changes in routines, dietary habits and food selection of the population.^13-39^ In our study, most of the participants stated that they had not changed their dietary habits; however, a total of 29.3% started eating more often, 33.3% stated increased consumption of sugary foods; 35% increased bakery food consumption; 34.7% increased meat and dairy product consumption, while vegetable and fruit consumption increased by 34.9% and 39.2%, respectively. The amount of energy and fat consumed in the diet increases under stressful conditions.^48^ Previous studies reported that eating frequency increased during the COVID-19 pandemic, consistent with our findings.^10,13^ In addition, a study stated that the consumption of carbohydrates increased during the COVID-19 pandemic.^39^ Dietary changes, economic problems, fear, and lack of preventive dental care are interrelated risk factors that can affect a person’s oral health.^10^ The negative effects of increased carbohydrate consumption on teeth during the pandemic process may appear in the future. While some studies, as opposed to our results, have reported that the consumption of fresh fruits and vegetables decreased,^6,44^ one study reported an increase in meat consumption,^9^ which was similar to our results. A study^13^ whose results were consistent with ours stated that during quarantine, those who attached importance to the Mediterranean diet in Italy increased, and the quality of nutrition improved. That study suggested increasing the intake of fruits and vegetables with high vitamin and antioxidant contents and a Mediterranean diet during the COVID-19 pandemic to strengthen immunity.^13^ Another study reported that participants turned directly to farmers or organic fruit and vegetables.^10^ A higher fear score was seen in our study among participants who ate more frequently, as well as those with increased consumption of sugary foods, bakery foods, meat and dairy products, and vegetables. Some studies^5,7^ stated that the inability to cope with fear and stress caused by COVID-19 leads to eating disorders and increased eating frequency. One study concluded that the COVID-19 epidemic negatively affected eating habits and dental care because of moderate and high levels of fear.^10^ These results are similar to our findings. A previous study found a relationship between increased stress and the intake of sweet and savory snacks, fast food, and cake-like foods.^47^ Additionally, stress-induced dietary preferences often include high amounts of added sugar or starch, which are referred to as comfort foods.^49^ Also, it was reported that families who have lost income ate less or chose cheaper foods during the pandemic.^10^ Foods with low nutritional value and high sugar could be among the more affordable foods. In our study, in accordance with these results, the tendency to consume carbohydrates in the diet increased with rising fear. The tendency to consume carbohydrate-rich foods not only affects overall health, but also increases the risk of developing caries and periodontal disease.

One of the main aims of our study was to evaluate how oral health changed during the COVID-19 pandemic and the fear of COVID-19 in individuals with dental complaints. To our knowledge, there is no study examining these in the literature. In our study, the fear of COVID-19 scores were found to be higher among participants who reported tooth hypersensitivity, toothache, swelling/abscess due to tooth decay, failure of restorations, prosthetic problems, periodontal tissue bleeding, periodontal pain, oral malodor, and bruxism. Fear of COVID-19 was 1.668 times higher and 1.941 times higher in participants with toothache upon heating hot food and bruxism, respectively, than in individuals without these complaints (OR: 1.668, 1.941; p = 0.045, 0.001, respectively). Studies have reported that bruxism has increased during the COVID-19 pandemic, and this situation may be due to the negative change in anxiety and psychosocial status due to COVID-19.^4,16^ According to our findings, it can be said that during the pandemic period, other complaints about oral and dental health may have increased due to changing nutrition and oral hygiene habits and fear of visiting a dental office/clinic due to their risk for virus contamination. In our study, the relationship between those whose dental complaints increased and those who had high fear of COVID-19 can be explained by restrictive conditions, such as participants’ hesitation to visit a dentist and being quarantined. Similarly, a study in the field of pediatric dentistry reported that children experienced dental trauma during the pandemic, but 86% of them did not receive dental care because of their parents’ fear of COVID-19.^10^ On the other hand, another study reported that regardless of whether the patients underwent dental treatment, they were anxious or afraid in relation to the COVID-19 pandemic.^38^ The restriction of access to dental clinics for different reasons, the increase in dental complaints during the pandemic, and its relationship with the fear of COVID-19 draws attention to the need for the regulation of preventive dentistry and dental treatment service policies during a pandemic.

Due to respiratory droplets, which are the main route of SARS-CoV-2 transmission, the risk of contamination with COVID-19 may be high between dental practitioners and patients during dental procedures, where a large number of droplets and aerosols are generated.^29^ One of the aims of our study was to evaluate the behaviour of people towards dental treatments due to the fear of COVID-19. Our study participants who had a dental problem but hesitated to see a dentist, those who became hesitant to go to the dentist compared to before the COVID-19 pandemic, and those who thought dental clinics were posed a risk for COVID-19 contamination had a higher fear score on the COVID-19 scale. Additionally, fear of COVID-19 was 1.946 times higher in participants who hesitated to see a dentist than in participants whose attitude in this regard had not changed (OR: 1.946, p = 0.001). In a study conducted in the early stages of the COVID-19 pandemic, the rate of emergency dental visits decreased by approximately 40%.^20^ In another survey study, Kranz et al^25^ reported that nearly half (46.7%) of the participants delayed going to the dentist or getting dental care due to the COVID-19 outbreak.^25^ Other studies, whose results are compatible with our findings, reported that patients who worried about COVID-19 were afraid of dental care and treatment, and most of participants thought any dental treatment would increase their risk of COVID-19 infection.^24-38^ A study similar to ours showed that people who value oral health are more cautious about going to the dentist.^30^ Additionally, individuals who brushed their teeth more regularly in our study had a higher fear of COVID-19 and avoided going to the dentist for treatment. Thus, for individuals to access dental services, it should be publicly broadcast that the necessary measures have been taken in dentistry to prevent the spread of infection.^30^ The use telemedicine tools and digital monitoring has been suggested to reduce repeated patient contacts and appointments to ensure patient safety.^28,45^

A possible limitation of the present study is that the survey was delivered to the participants via e-mail and WhatsApp; the latter requires use of a smartphone. Therefore, mostly young people participated in the study, and participants was lower among older age groups. Likewise, the respondents of the survey are mostly individuals with high economic and educational status. Another limitation of this study is its cross-sectional design. Further longitudinal studies that will guide policy on providing information about preventing excessive fear, enabling access to dental services, and preventing risk factors during epidemics are needed.

Our study provides some information about individuals’ knowledge, awareness level, perception, and attitudes during the pandemic process. According to the findings of the present study, attention can be drawn to the necessity of determining the fear levels of individuals in possible pandemic processes, alleviating their fears, revealing their nutritional and oral hygiene needs, and providing the information required to facilitate access to dental health services. Additionally, the present findings provide data on the changes in routine life, oral care, dental services, and health perceptions that occur due to people’s fear of COVID-19, thus raising awareness and helping provide a basis for new regulations.

## CONCLUSIONS

The present study indicated an association between COVID-19 fear and oral health status. Higher fear of COVID-19 was observed in participants who began to pay more attention to their oral hygiene habits, with changes in their frequency of food consumption and in the consumption rates of certain foods. In addition, higher fear was observed in individuals with increased complaints about oral and dental health, who had a dental problem but hesitated to see a dentist, and who believed that dental clinics put them at risk of being infected with COVID-19.
